# Enterococcal PcfF Is a Ribbon-Helix-Helix Protein That Recruits the Relaxase PcfG Through Binding and Bending of the *oriT* Sequence

**DOI:** 10.3389/fmicb.2019.00958

**Published:** 2019-05-07

**Authors:** Saima Rehman, Yang Grace Li, Andreas Schmitt, Lena Lassinantti, Peter J. Christie, Ronnie P.-A. Berntsson

**Affiliations:** ^1^Department of Medical Biochemistry and Biophysics, Umeå University, Umeå, Sweden; ^2^Department of Microbiology and Molecular Genetics, McGovern Medical School, Houston, TX, United States; ^3^Wallenberg Centre for Molecular Medicine, Umeå University, Umeå, Sweden

**Keywords:** T4SS, accessory factor, conjugation, relaxosome, X-ray crystallography, protein structural and functional analysis

## Abstract

The conjugative plasmid pCF10 from *Enterococcus faecalis* encodes a Type 4 Secretion System required for plasmid transfer. The accessory factor PcfF and relaxase PcfG initiate pCF10 transfer by forming the catalytically active relaxosome at the plasmid’s origin-of-transfer (*oriT*) sequence. Here, we report the crystal structure of the homo-dimeric PcfF, composed of an N-terminal DNA binding Ribbon-Helix-Helix (RHH) domain and a C-terminal stalk domain. We identified key residues in the RHH domain that are responsible for binding pCF10’s *oriT* sequence *in vitro*, and further showed that PcfF bends the DNA upon *oriT* binding. By mutational analysis and pull-down experiments, we identified residues in the stalk domain that contribute to interaction with PcfG. PcfF variant proteins defective in *oriT* or PcfG binding attenuated plasmid transfer *in vivo*, but also suggested that intrinsic or extrinsic factors might modulate relaxosome assembly. We propose that PcfF initiates relaxosome assembly by binding *oriT* and inducing DNA bending, which serves to recruit PcfG as well as extrinsic factors necessary for optimal plasmid processing and engagement with the pCF10 transfer machine.

## Introduction

*Enterococcus faecalis* can transfer pheromone-inducible plasmids in a highly efficient manner upon sensing the peptide pheromones produced by recipient cells. These plasmids encode three functional modules of importance for plasmid transfer: (i) the Dtr (DNA transfer and replication) proteins responsible for processing of the plasmid for transfer, (ii) the Mpf (mating-pair formation) proteins that assemble as the translocation channel or type IV secretion system (T4SS), and (iii) cell-wall anchored adhesins that facilitate formation of donor-recipient cell mating pairs (Alvarez-Martinez and Christie, 2009). Over the past decade, studies have advanced our understanding of the mechanisms of action and structures of T4SSs and Dtr factors functioning in Gram-negative (G^−^) species (Grohmann et al., 2017). Systems functioning in Gram-positive (G^+^) species, however, remain less well-understood. While some mechanistic and architectural features are likely conserved among all conjugative machines, key steps of substrate processing and recruitment, mating pair formation, and substrate transfer can be expected to differ substantially between systems functioning in diderm vs. monoderm species (Bhatty et al., 2013; Grohmann et al., 2017).

The tetracycline-resistance plasmid pCF10 from *E. faecalis* is a member of the highly transmissible pheromone-responsive family of mobile genetic elements (MGEs) found in enterococci. The encoded T4SSs of these pheromone regulated MGEs are tightly regulated at the transcriptional level by sensing of peptide pheromones originating from recipient cells (Dunny, 2013; Dunny and Berntsson, 2016). The broad medical importance of this large family of pheromone-inducible plasmids is underscored by the fact that they serve as reservoirs for genes encoding many different virulence factors, adhesins and antibiotic resistance. Additionally, they can mobilize other MGEs to both enterococcal and non-enterococcal recipients (Antiporta and Dunny, 2002; Staddon et al., 2006).

In this study we focused on two of the Dtr proteins, the PcfF accessory factor and the PcfG relaxase. PcfF binds to double stranded DNA (dsDNA) and is specific for inverted repeat sequences located within pCF10’s origin of transfer (*oriT*) sequence (Chen et al., 2007). PcfG exhibits no intrinsic affinity for the *oriT* sequence, or any dsDNA, in the absence of PcfF. PcfF-*oriT* complexes, however, recruit PcfG to form the relaxosome, as evidenced by supershifting of PcfF-*oriT* complexes in the presence of PcfG to higher molecular mass complexes in electrophoretic mobility shift assay (EMSA) experiments (Chen et al., 2007). PcfG then catalyzes strand-specific nicking at *oriT* and generation of the single-stranded transfer intermediate (T-strand) (Chen et al., 2007, 2008; Li et al., 2012). After cleaving the substrate, PcfG remains covalently bound to the 5′ end of the T strand and likely pilots it through the conjugation channel and into the recipient cell, as has been shown for relaxases functioning in G^−^ systems (Alvarez-Martinez and Christie, 2009). PcfG also catalyzes the re-joining of cleaved *nic* sites *in vitro*, a reaction thought to direct T strand re-circularization, second-strand synthesis and plasmid stabilization in the recipient cell (Chen et al., 2007; Alvarez-Martinez and Christie, 2009).

Some G^−^ accessory factors have been well studied, including the TraM and TraY proteins of the F plasmid, MbeC from the ColE1 plasmid, TrwA of the R388 plasmid and NikA from the R64 plasmid (Luo et al., 1994; Moncalian and de la Cruz, 2004; Yoshida et al., 2008; Varsaki et al., 2009; Wong et al., 2011). These proteins have all been shown to contain a Ribbon-Helix-Helix (RHH) domain responsible for binding to DNA. RHH domains are a well-characterized family of transcriptional repressors in bacteria, first characterized with the bacterial MetJ and Arc repressors (Somers and Phillips, 1992; Raumann et al., 1994; Schreiter and Drennan, 2007). In contrast to the common helix-turn-helix motif for DNA binding, RHH domains bind DNA via a small N-proximal β-sheet composed of one β-strand from each monomer (Schreiter and Drennan, 2007). So far, no accessory factors of G^+^ origin have been structurally characterized, albeit one *in silico* analysis has indicated that many of them also contain RHH-binding domains (Miguel-Arribas et al., 2017).

Here, we determined the structure of PcfF and show that it contains an N-terminal RHH domain and a C-terminal stalk domain. PcfF is a dimer in solution and structure-guided mutational analyses identified residues involved in DNA binding and residues required for interaction with PcfG. Together, our findings expand our knowledge of how accessory factors coordinate assembly of the relaxosome in G^+^ bacteria. They also suggest the importance of other intrinsic, e.g., DNA bending, and extrinsic factors for relaxosome assembly *in vivo*.

## Experimental Procedures

### Bacterial Strains and Plasmids

The *Escherichia coli* pET plasmid pCY33 carrying wild-type *pcfF* listed in Supplementary Table S1 were used to generate plasmids harboring the following *pcfF* mutations: R13L (pYGL194), R13L/I14A (pYGL196), I70S (pYGL197), 1-54 (pYGL199). Mutated genes were confirmed by sequencing using the T7F primer. The *pcfF* alleles on pYGL194, 196, 197, and 199 were then amplified with primers XhoI_pcfF_F and SphI_pcfF_R, the PCR products were digested with XhoI and SphI, and the digested products were introduced into similarly digested pDL278p23 to generate plasmids carrying the *pcfF* variants: R13L (pYGL202), R13L/I14A (pYGL203), I70S (pYGL205), 1-54 (pYGL205). Constructs were confirmed by sequencing with the M13F primer. These plasmids introduced by electroporation into *E. faecalis* strain CK104 (pCF10Δ*pcfF*).

For protein production, *pcfF* (GeneBank accession AAW51324) was PCR amplified using pCF10 as a template and cloned into pGEX-6P-2 using BamHI/XhoI. The truncated version PcfF_1–54_ (lacking residues 55–118) was made by mutation of Tyr 55 to a stop codon. QuikChange mutagenesis was used to generate single, double, and triple mutations of *pcfF* (R13L, I14A, R16L, R13L/I14A, R13L/R16L, R13L/I14A/R16L, I70S, N73A/Q74A, R77S, I70S/R77S, Q105A/W) with *pcfF* expression vectors as templates. *pcfG* (GeneBank accession AAW51325) was PCR amplified from pCF10 and inserted into pBAD expression vectors via the FX cloning system (Geertsma and Dutzler, 2011).

### Protein Expression and Purification

PcfG (with a C-terminal deca-histidine tag), PcfF and variants thereof (all with a N-terminal GST tag) were produced in *E. coli* BL21(DE3). For PcfF and the variants of PcfF the cells were grown at 37°C in 2 × YT medium until they reached an OD_600_ of ca 1.0. At that time, the temperature was lowered to 18°C and expression was induced by the addition of 0.4 mM IPTG. Cells were grown for 16 h before harvesting. Production of selenomethionine derivatized PcfF was carried out in *E. coli* BL21(DE3) grown in M9 minimal media supplemented with 50 mg/mL L-Selenomethionine as described previously (Vanduyne et al., 1993). Derivatized PcfF was purified as described below for wild type PcfF, with the addition of 0.5 mM TCEP [tris(2-carboxyethyl)-phosphine] to all buffers after affinity purification. PcfG was produced in the same way as PcfF, with the exception that TB medium was used instead of 2 × YT and that the cells were induced by the addition of 10^−2^% (w/v) L-arabinose at an OD_600_ of 0.8. The cells were resuspended in lysis buffer (20 mM Tris/HCl pH 7.5, 300 mM NaCl, 0.02 mg/ml DNase I, and 0.5 mM proteinase inhibitor AEBSF) before disrupting them using a Constant Cell Disruptor (Constant Systems) at 25 kPsi and 4°C. Cell debris was removed by centrifugation at 16000 × *g* for 15 min.

Wild type and variant forms of GST-PcfF were incubated for 1 h at 4°C with Glutathione-Agarose bead (Protino). The beads were packed in a gravity flow column and washed with 20 mM Tris/HCl pH 7.5, 200 mM NaCl, before eluting the protein with elution buffer (20 mM Tris/HCl pH 8.0, 200 mM NaCl, 30 mM reduced Glutathione, and 10% Glycerol). Subsequently, the proteins were run on a Superdex 200 10/300 GL Increase column (GE Healthcare) in SEC buffer (20 mM Tris/HCl pH 7.5, 200 mM NaCl). For GST-tag cleavage, 3C protease was added in a ratio of 1:100 and incubated for 16 h at 4°C. To isolate the cleaved sample a second gel-filtration step was performed in SEC buffer. The free-GST tag coeluted with cleaved PcfF, and therefore reverse-purification was performed by passing the purified sample through pre-equilibrated Glutathione-Agarose beads. The single, double and triple variants of PcfF (R13L, I14A, R16L, R13L/I14A, R13L/R16L, R13L/I14A/R16L, I70S, N73A/Q74A, R77S, I70S/R77S, Q105A, and Q105W) all behaved virtually the same as wild type PcfF, with identical elution volume on the SEC. PcfF_1–54_ has a molecular mass of 7 kDa for a monomer (PcfF residues 1-54 plus 4 extra residues left on after cleaving off the GST). PcfF_1–54_ eluted with an apparent molecular mass of 17 kDa on a Superdex 75 10/300 GL column. Due to the absence of Trp in PcfF, the concentration of the protein was determined using a BCA assay (Pierce).

PcfG-His was purified via refolding as previously described (Chen et al., 2007). Briefly, the protein was dissolved in binding buffer (8 M Urea, 20 mM Tris/HCl pH 7.5, 200 mM NaCl, 15 mM Imidazole pH 7.8) and bound to Ni-NTA sepharose beads (Macherey-Nagel) at 4°C. The column was washed with 10 column volumes (CV) wash buffer (8 M Urea, 20 mM Tris/HCl pH 7.5, 200 mM NaCl, and 50 mM Imidazole pH 7.8) before being eluted. PcfG-His was then refolded via a 4-step dialysis to decrease the Urea and Imidazole concentrations. The refolded PcfG-His was subsequently run on a Superdex 200 10/300 GL Increase column, where the protein eluted at the expected volume for a monomer.

### Gas-Phase Electrophoretic Mobility Macromolecule Analysis

Gas-phase electrophoretic mobility macromolecule analysis (GEMMA) on PcfF was performed as previously described (Rofougaran et al., 2008). Briefly, the peak of wild type PcfF from size exclusion chromatography (SEC) was dialyzed against 100 mM ammonium acetate, pH 7.8 and then diluted to a concentration of 0.025 mg/mL in a buffer containing 100 mM ammonium acetate, pH 7.8 and 0.005% Tween-20. This protein sample was then analyzed by GEMMA.

### Crystallization and Structure Determination

Crystals of selenomethionine incorporated PcfF were grown at 20°C by sitting drop vapor diffusion in a condition containing 0.2 M Lithium sulfate, 0.1 M Bis-Tris pH 6.5, 25% (w/v) PEG 3350 with a protein concentration of 16 mg/mL and a protein:reservoir ratio of 1:1. Crystals were flash-frozen in liquid nitrogen without additional cryo-protectant. X-ray diffraction data of SeMet-PcfF was collected on ID30A-3 ESRF, France at the selenium edge. The data were processed using XDS (Kabsch, 2010). The crystallographic phase-problem was solved using the single anomalous diffraction data and the selenium sites were found and refined by the Auto-Rickshaw software (Panjikar et al., 2005) with an initial model being built by ARP/wARP (Cohen et al., 2008). The PcfF crystals belonged to space group P2_1_ and contained 4 molecules in the asymmetric unit. The structure was further built in Coot and refined at 1.9 Å using PHENIX refine (Adams et al., 2002; Emsley and Cowtan, 2004), to R_work_/R_free_ values of 19.0/23.0%. For complete data collection and refinement statistics see Supplementary Table S2. The structure has been deposited in the Protein Data Band (PDB code: 6QEQ).

### Electrophoretic Mobility Shift Assay

Electrophoretic mobility shift assays were performed as described elsewhere (Hellman and Fried, 2007). Different versions of single stranded *oriT* DNA were purchased from Eurofins Genomics, with the sense strand labeled at the 5′ end with fluorescein isothiocyanate (FITC). The sequences of different DNA segments used in this study are presented in Supplementary Table S3. Double-stranded DNA was obtained by mixing equimolar concentration of the sense and antisense strand in annealing buffer (10 mM Tris, 1 mM EDTA, 50 mM NaCl, pH 8.0). The annealing reaction was carried out by incubation at 94°C for 2 min followed by gradual cooling. For further purification, the annealed DNA was run on a 20% polyacrylamide gel, the band containing the fluorescent double strand was excised and the DNA eluted from the gel. Duplex *oriT* DNA (30 nM) was mixed with increasing concentrations of PcfF between 0 to 960 nM in buffer containing 10 mM Tris/HCL pH 7.5, 200 mM NaCl. For the binding studies with PcfF and PcfG, 30 nM of DNA, 100 nM of the PcfF variants and 300 nM of PcfG were mixed in 10 mM Tris/HCL pH 7.5, 150 mM NaCl. The DNA-protein mixtures were incubated at room temperature for 15 min and subsequently loaded on to a 20% native-PAGE made in TAE buffer (40 mM Tris, 20 mM acetic acid, 1 mM EDTA, pH 8.0). Electrophoresis was carried out for 70 min at 110 V and 4°C. The gel was imaged on a Typhoon scanner. The bands were visualized using a 488 nm excitation filter. ImageQuant software was used to quantify the fluorescent signal of the bands, and the curves from the resulting data were fitted to a non-linear fit (Specific binding with Hill slope) using GraphPad Prism.

### DNA Bending Assay

Purified PcfF/PcfF_1–54_ and 120 bp long DNA fragments, each containing the *oriT* sequence at varying positions (Supplementary Table S3) were mixed in 20 μl of 10 mM Tris/HCl (pH 7.5), 150 mM NaCl to a final protein and DNA concentration of 300 and 30 nM, respectively. After incubation at room temperature for 20 min, the reaction mixtures were loaded onto a 5% (w/v) polyacrylamide gel (acrylamide/bisacrylamide in a ratio of 37.5:1. w/w) in buffer (40 mM Tris–acetate (pH 7.8), 1 mM EDTA) and were electrophoresed at 10 V/cm and 4°C. Following electrophoresis, the DNA fragments in the gel were stained with GelRed and visualized under UV light.

### *In vitro* Pull-Down Assay

For GST-pull-down experiments, 2 nmol of GST-PcfF fusion protein (or variants thereof) were immobilized on GSH-Sepharose beads, while 4 nmol PcfG-His were used as pray protein. Purified proteins were incubated with pre-equilibrated 50 μl Protino^®^ Glutathione-Agarose 4B beads. BSA and purified GST were used as controls. All proteins were dialyzed against the same buffer (20 mM Tris/HCl pH 7.5, 200 mM NaCl). After binding, the beads were washed extensively (5 × 10 CV) and subsequently eluted by elution buffer (20 mM Tris/HCl pH 8.0, 200 mM NaCl, 30 mM reduced Glutathione). Samples from wash and elution steps were analyzed on 15% SDS-PAGE and stained with Coomassie Brilliant Blue G-250.

### Detection of PcfF Mutant Proteins in *E. faecalis*

Exponential-phase cultures (10 ml) of *E. faecalis* OG1RF strains carrying pCF10Δ*pcfF* without and with plasmids producing wild type or mutant PcfF proteins were normalized to an OD_600_ of 0.3. The cells were pelleted by centrifugation at 13,200 × *g* for 15 min at 4°C and washed once with cold 1X physiological buffer saline (PBS). The pellet was resuspended in 125 μl of SMM buffer (0.5 M sucrose, 0.02 M MgCl_2_, 0.02 M maleate, pH 6.5) containing 60 μl ml^−1^ of mutanolysin (Sigma-Aldrich) and 10 mg ml^−1^ of lysozyme (Sigma-Aldrich), and the resulting mix was incubated for 1 h at 37°C with shaking. Material released from the digested cell wall was separated from cell-bound material by centrifugation at 13.200 × *g* for 15 min at 4°C. PcfF variants were detected by Western transfer and immunostaining with the anti-PcfF antibodies (Chen et al., 2008). Blots were probed with antibodies against the β-subunit of RNA polymerase as a protein loading control (Christie et al., 1988; Chen et al., 2007, 2008).

### Conjugation Assays

*Enterococcus faecalis* donor and recipient cultures grown overnight were diluted 1:10 in BHI (Brain Heart Infusion broth; Sigma) and incubated for 1 h at 37°C without shaking. Donor and recipient cells were mixed in a ratio of 1:1 and allowed to mate in liquid without shaking for 1 h at 37°C. Mating mixtures were serially diluted in BHI, and the numbers of donors and transconjugants were obtained by plating on selective BHI agar plates. The plasmid transfer frequencies were calculated as the number of transconjugants per donor cell (Chen et al., 2008). The results are reported as an average of three replicates of each experiment.

## Results

### PcfF Is a Dimer in Solution

**FIGURE 1:**
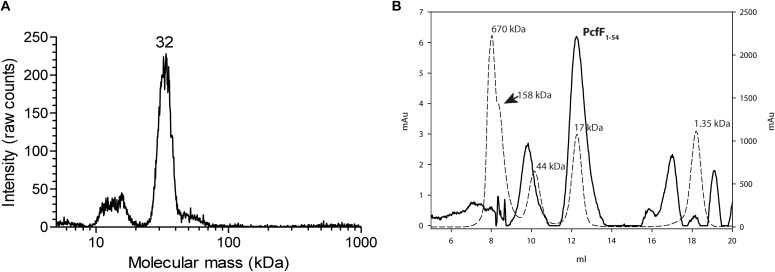
Oligomeric state of PcfF. **(A)**: GEMMA analysis of PcfF. The sample was taken from the elution peak of the size exclusion chromatography fraction. The GEMMA analysis was performed with a protein concentration of 0.025 mg/mL. The determined molecular masses (in kDa) are written above the peak. **(B)**: SEC analysis of PcfF_1–54_ on a Superdex 75 10/300 GL column, with molecular mass standards as dashed lines and PcfF_1–54_ as a solid line. The molecular mass in kDa is written above each standard in the graph. Since PcfF_1–54_ does not have any Tryptophan residues, the left Y axis denotes the absorbance at 254 nm for PcfF_1–54_, while the right y-axis shows the absorbance at 280 nm for the standards. The main peak for PcfF_1–54_ elutes at 17 kDa, with the earlier peak corresponding to free GST after the cleavage of the protein as confirmed by SDS-PAGE analysis.

Full-length PcfF (14 kDa) was produced in *E. coli* and purified to homogeneity. It eluted as a single peak on SEC, with an apparent molecular mass of ∼48 kDa. The molecular mass of wild type PcfF from the SEC peak was determined by gas-phase electrophoretic mobility macromolecule analysis (GEMMA, also termed Macroion mobility spectrometer) to be ∼32 kDa, very close an apparent dimer (Figure 1A; Kaufman et al., 1996; Bacher et al., 2001). PcfF_Q105A/W_ variants that were made to probe potential differences in oligomerization state, showed no difference in elution volume on SEC as compared to wt PcfF. PcfF_1–54_ eluted as a single peak on SEC, with an apparent molecular weight of 17 kDa (Figure 1B), close to the expected 14 kDa weight of a dimer.

We next crystallized full-length selenomethionine-incorporated PcfF for structural analysis. Crystals belonged to space group P2_1_ and contained 4 molecules in the asymmetric unit. X-ray diffraction data was collected at the selenium edge and the phase problem solved by SAD phasing (Supplementary Table S2). The structure was refined at a resolution of 1.9 Å. The electron density accounted for the entire PcfF protein, with the exception of 1–7 residues that were missing at the N terminus and one residue at the C terminus, the exact number varying between each of the 4 protein chains in the structure. PcfF crystallized as a tetramer (dimer of dimers in a head to toe organization) (Supplementary Figure S1). However, the tetrameric interface is weak and deemed unstable by PISA calculations (Krissinel, 2015). The biologically relevant oligomer was suggested to be dimeric, in agreement with results from the SEC and GEMMA experiments. The dimeric structure is extended in one dimension, which explains why in size exclusion chromatography PcfF elutes at a higher apparent molecular mass than expected. Functional analysis described in the next section also points toward PcfF functioning as a dimer. From here onward, we base our structural analysis on one of the dimers, made up by chains A and C.

### PcfF Contains a DNA-Binding RHH Domain

**FIGURE 2:**
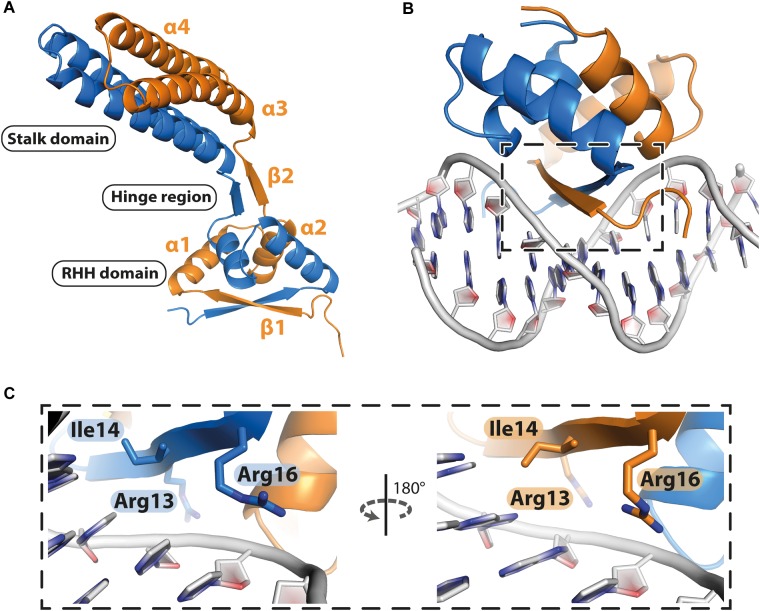
Structure of PcfF. **(A)**: Overall representation of the dimeric PcfF structure, with the RHH and stalk domains connected by a hinge region. Both monomers contribute to the formation of the RHH domain. **(B)**: Model of PcfF with bound DNA, based on superposition with ArcA structure (Supplementary Figure S2). **(C)**: Enlarged view of the box from panel **B**, with the conserved DNA binding residues R13, I14, and R16 highlighted as sticks.

**FIGURE 3:**
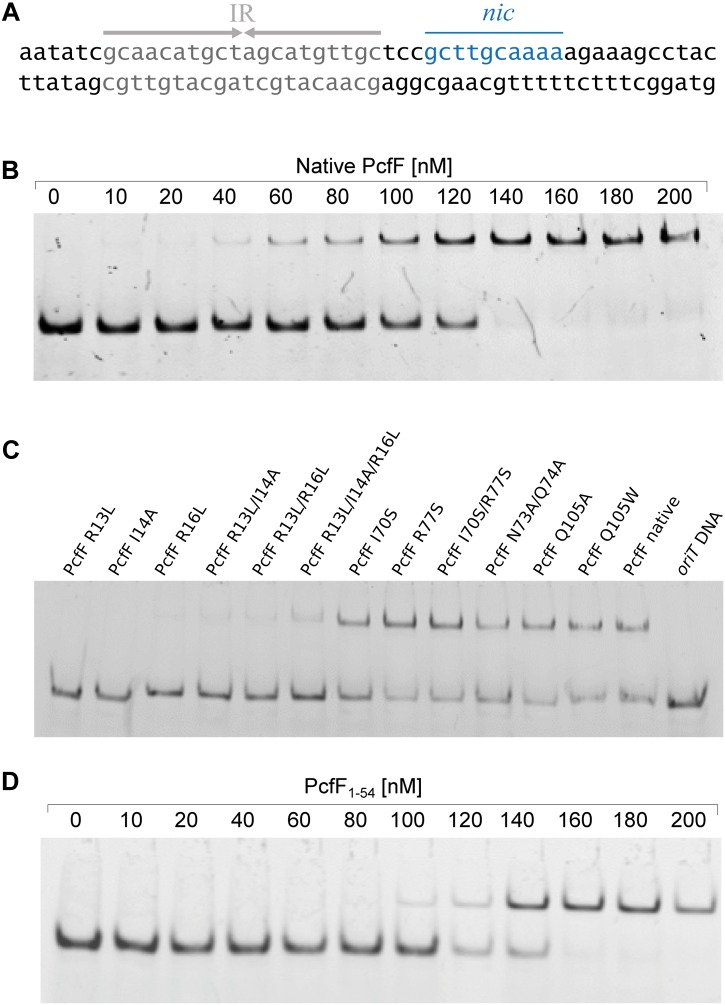
EMSA of PcfF variants binding to *oriT.* In all cases the 30 nM of the 40 bp long double stranded *oriT* substrate was used. **(A)**: Sequence of the *oriT* (40 bp) from pCF10. The inverted repeats (IR), which PcfF binds to, and the nic-site are highlighted. **(B)**: Wild type PcfF binds to a 40 bp *oriT* substrate. The increasing concentrations of PcfF added are indicated above the lanes. **(C)**: PcfF variants bind with varying degrees to the 40 bp *oriT* sequence. Reaction mixtures contained 100 nM PcfF. **(D)**: PcfF_1–54_ binds to *oriT*. The increasing concentrations of PcfF added are indicated above the gel. The reactions were analyzed on 20% native polyacrylamide gels. Protein components in each lane are shown on the top of the lane.

PcfF contains a RHH domain at the N terminus and a 2-helix bundle, here termed the stalk domain, at the C terminus. These two domains are connected by a hinge region (Figure 2A). In the dimer, the RHH and stalk domains are built up by secondary structure elements from both monomers in the dimer. By superimposition of PcfF’s RHH domain on other RHH motifs associated with DNA, we determined that 3 residues, R13, I14, and R16, likely are involved in DNA binding (Figure 2B,C and Supplementary Figure S2). These residues were mutated and effects on DNA binding were assessed using an EMSA. Wild type PcfF bound a 40 bp *oriT* sequence, composed of double-stranded inverted repeats and the *nic*-site, with an estimated K_D_ of ∼100 nM, in agreement with previous findings (Chen et al., 2007; Figure 3A,B). PcfF did not bind a random DNA sequence, verifying a specificity for *oriT* binding (Supplementary Figure S3). PcfF variants with single (R13L, I14A, R16L), double (R13L/I14A, R13L/R16L), or triple (R13L/I14A/R16L) substitution mutations showed marked decreases in *oriT* binding (Figure 3C). We also confirmed that the RHH domain (PcfF_1–54_) without the associated stalk domain bound *oriT* with little reduction in affinity (Figure 3D).

### The Stalk Domain of PcfF Binds PcfG

**FIGURE 4:**
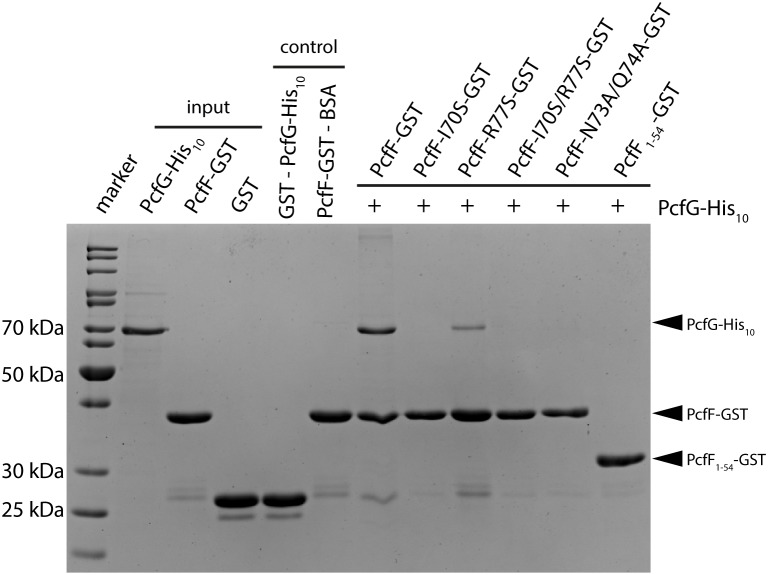
SDS-PAGE analysis of GST pull-down of PcfG. GST-tagged PcfF and variants thereof were compared for their ability to bind PcfG-His_10_. As controls, GST alone was assayed for binding of PcfG-His_10_, and PcfF-GST was assayed for binding of BSA. Protein bands were visualized by staining with Coomassie Blue.

PcfF binds the relaxase PcfG, as determined by EMSAs and affinity pull-down assays (Chen et al., 2007, 2008). PcfF possesses a sequence-motif NINQ in a surface-exposed region of the C-terminal stalk; this motif is semi-conserved among other T4SSs accessory proteins associated with conjugation systems in Gram-negative and -positive bacteria (Supplementary Figure S4A) (Varsaki et al., 2009). In the PcfF X-ray structure, the NINQ motif forms a patch within a small groove (Supplementary Figure S4B). We hypothesized that this motif might comprise the binding surface for PcfG. To test this model, we introduced several mutations (I70S, R77S, I70S/R77S, N73A/Q74A) in and around this conserved surface patch. These PcfF variants, as well as PcfF_1–54_ lacking the entire stalk domain, behaved as wild type PcfF with respect to purification as dimers and binding of *oriT* DNA (Figure 3C,D). We next tested for effects of the mutations on PcfF binding to PcfG using affinity pull-down assays. GST-PcfF and variants thereof were incubated with PcfG-His and Glutathione-Agarose beads. Following extensive washing, proteins were eluted and analyzed for the presence of GST-PcfF and PcfG-His. As shown previously, wild type GST-PcfF pulled down PcfG-His (Figure 4; Chen et al., 2008). None of the GST-PcfF variants detectably bound PcfG-His, except for the R77S mutant which showed a low level of binding. We further assayed for the ability of PcfG-His to bind to PcfF-*oriT* or PcfF_1–54_-*oriT* complexes via EMSAs, but did not observe any additional supershifted bands upon the addition of PcfG-His (Supplementary Figure S5).

### PcfF Binding Induces DNA Bending

Other T4SS accessory factors with RHH domains have been shown to induce bending of DNA (Yoshida et al., 2008; Wong et al., 2011). To determine if PcfF induces a bend in the pCF10 *oriT* sequence, we capitalized on findings that bent DNA fragments exhibit an anomalous electrophoretic mobility behavior, which is most pronounced when the bending locus is located close to the center of the fragment (Thompson and Landy, 1988; Levene and Zimm, 1989). We performed these DNA bending experiments using 120 bp fragments of random DNA with the *oriT* positioned at the 5′ end, middle or 3′ end of the DNA (Supplementary Table S3) (Crothers et al., 1991). Binding of PcfF induced a more pronounced shift in the DNA fragment containing the central *oriT* sequence compared with fragments in which *oriT* was positioned at either end. These findings support a conclusion that PcfF does indeed induce bending of DNA at the *oriT* site (Supplementary Figure S6). A similar trend could be seen for PcfF_1–54_ as for PcfF. However, for unknown reasons, PcfF_1–54_ bound to the 120 bp long DNA yielded smeary band shifts and therefore prevent firm conclusions regarding the capacity of PcfF’s RHH domain to induce DNA bending.

### PcfF DNA Binding and Relaxase Recruitment Is Not Essential for Conjugation *in vivo*

**FIGURE 5:**
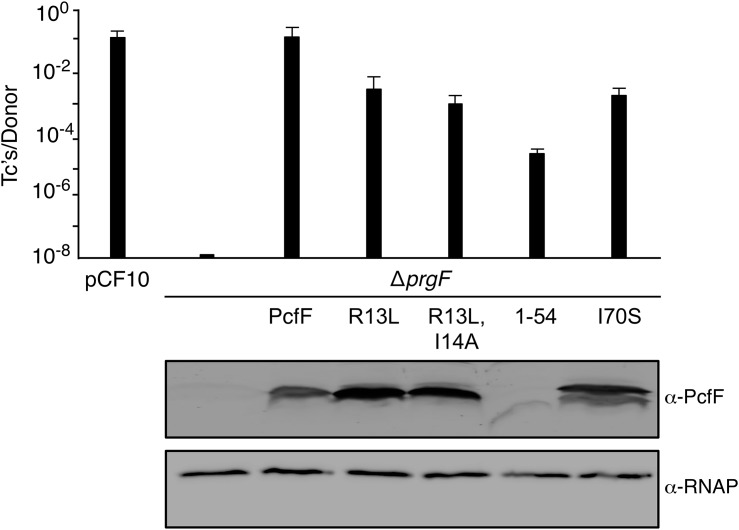
Effects of PcfF mutations on pCF10 transfer. Upper graph: CK104 donors harboring pCF10Δ*pcfF* and producing wild type and mutant variants of PcfF from the constitutive P_23_ promoter were mated with OG1ES recipients for 1 h in BHI media at 37°C without shaking. Transfer frequencies are presented as the number of transconjugants (Tc’s) per donor. Experiments were repeated at least three times in triplicate, and results from a representative experiment with standard deviations are shown. Lower panels: Steady-state levels of PcfF proteins in exponentially growing strains. Immunoblots were developed with α-PcfF antibodies for detection of PcfF variants or α-RNAP antibodies for detection of the β-subunit of RNA polymerase as a loading control. Protein extracts were loaded on a per-cell equivalent basis. Strains for both panels: CK104 (pCF10ΔpcfF) alone (ΔpcfF) or with plasmids producing PcfF (pCY33), PcfF_R13L_ (pYGL202), PcfF_R13L/I14A_ (pYGL203), PcfF_1–54_ (pYGL204), pcfF_I70S_ (pYGL205).

Finally, we determined the effects of the PcfF mutations on pCF10 transfer *in vivo* (Figure 5). *E. faecalis* strain CK104(pCF10D*pcfF*) does not transfer the mutant plasmid unless it additionally carries pCY16, which produces wild type PcfF from the constitutive P_23_ promoter. Interestingly, CK104(pCF10D*pcfF*) harboring plasmids producing the RHH mutant proteins PcfF_R13L_ and PcfF_R13L/I14A_ which fail to bind *oriT* DNA *in vitro*, also transferred pCF10D*pcfF* albeit at reduced frequencies of 1 to 2 orders of magnitude compared with CK104(pCF10D*pcfF*, pCY16). Similarly, the CK104(pCF10D*pcfF*) donor with plasmids producing variants defective in binding PcfG *in vitro* (PcfF_1–54_, PcfF_I70S_) mutant also were transfer-proficient, although at reduced levels. In CK104 donor strains, the full-length mutant proteins accumulated at levels comparable to or even higher than wild type PcfF. PcfF_1–54_ was detected at low levels, possibly reflecting instability or poor recognition by the anti-PcfF polyclonal antibodies. Formation of the PcfF/PcfG/*oriT* relaxosome on pCF10 thus appears to depend not only on PcfF residues responsible for binding *oriT* and PcfG *in vitro*, but on DNA structures formed *in vivo* or other unidentified host-encoded factors.

## Discussion

Conjugative transfer of MGEs happens by: (i) assembly of the relaxosome at *oriT* sequences, (ii) relaxase-catalyzed nicking of the DNA strand destined for transfer (T-strand), (iii) relaxosome recruitment to the type IV coupling protein (T4CP), and (iv) translocation of the relaxase/T-strand intermediate through the transfer channel (Alvarez-Martinez and Christie, 2009; Wong et al., 2012; Grohmann et al., 2017). Dtr accessory factors are known to be required for assembly of the relaxosome, but in most cases the molecular details surrounding this early stage reaction are unknown. Here, we have solved the structure of the accessory factor PcfF, which binds the pCF10 *oriT* sequence and recruits the PcfG relaxase for relaxosome assembly. Like several other accessory factors, albeit far from all, PcfF is essential for conjugation. We showed that PcfF is composed of an N-terminal RHH domain and a C-terminal a-helical stalk domain. Although residues in both of these domains contribute to dimerization of PcfF, the RHH domain also dimerizes in the absence of the stalk domain. We further confirmed structure-based predictions that β-strands within PcfF’s RHH domain contribute to *oriT* binding and gained evidence that a specific patch on the C-terminal stalk of PcfF mediates binding of PcfG.

**FIGURE 6:**
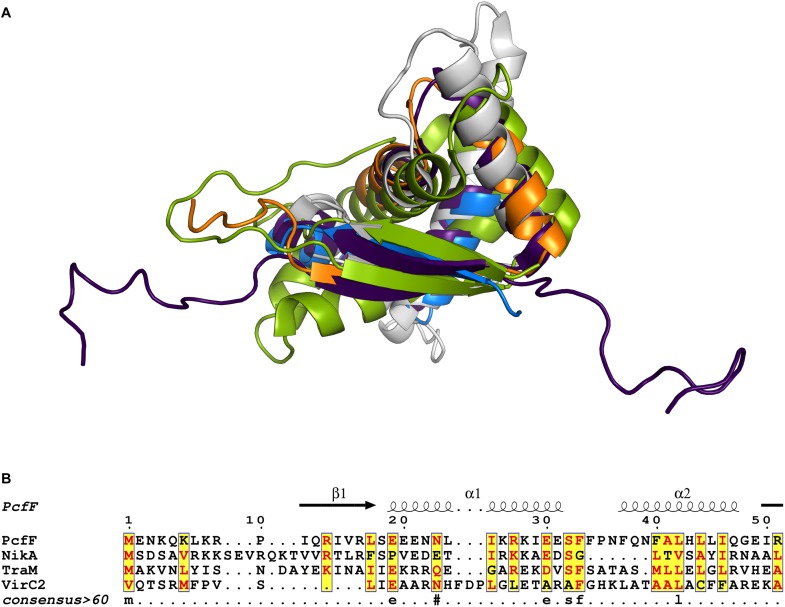
Structural and sequence comparison of four T4SS accessory proteins. **(A)** Superimposed structures of the RHH domain of PcfF (blue and orange), TraM (PDB code 3ON0, gray), NikA (PDB code 2BA3, purple), and VirC2 (PDB code 2RH3, green). PcfF and NikA have similar overall RHH domain structures, with RMSD of ca 1.5 Å. TraM and VirC2 vary more with RMSDs of ca 4.5 Å, but clearly share the same overall fold. **(B)** Sequence alignment of the RHH domains of the four proteins, with the secondary structure elements indicated above the alignment. The alignment file was generated through T-COFFEE server, followed by ESPript3 for rendering alignments (Armougom et al., 2006; Robert and Gouet, 2014). Text in red denotes conserved residues within a group. Blue frame with yellow background highlights similarity across groups and lowercase character denotes the consensus residue for consensus level >0.6.

Prior to this study, only three T4SS encoded accessory factors (TraM, NikA, and VirC2) have been structurally determined to our knowledge (Yoshida et al., 2008; Lu et al., 2009; Wong et al., 2011). Each belong to the RHH superfamily whose members are best known as bacterial transcription factors. Superimposition of these proteins with PcfF reveal that their RHH domains have overall similar structures, with RMSD values of 1.5 – 4 Å (Figure 6A). These proteins bind DNA through intercalation of their small two-stranded β-sheet within the RHH domain into the major groove of double stranded DNA; this interaction contributes both to affinity and specificity of DNA substrate binding (Schreiter and Drennan, 2007). Structurally, this β-sheet is made up by the first β-strand of each monomer in the RHH domain. Within this β-strand, a few structurally equivalent residues are (semi)conserved among the various accessory factors (Figure 6B). These residues have been implicated to be important for DNA binding, as established by solved structures of TraM or ArcA bound to DNA substrates (Raumann et al., 1994; Wong et al., 2011). In agreement with findings from other RHH accessory factors, we determined that PcfF binds pCF10’s *oriT* sequence via its RHH-domain, and that mutation of the surface-exposed charged residues R13 and R16, as well as I14, in the β-sheet strongly abrogate *oriT* binding *in vitro*. Deletion of the stalk domain does not impair *oriT* binding, confirming the RHH domain is both necessary and sufficient for binding the DNA substrate.

F plasmid-encoded TraM and another RHH domain-containing protein, TraY, bend and induce localized denaturation upon binding of DNA substrates (Luo et al., 1994; Karl et al., 2001; Fekete and Frost, 2002). Like PcfF, TraM comprises an RHH- and a C-terminal stalk domain. However, in contrast to what seems to be the case for PcfF, TraM assembles as a tetramer with two RHH domains and one larger 8-helical bundle stalk domain forming the tetramerization interface (Supplementary Figure S7). TraM binds to its cognate DNA cooperatively, which induces DNA bending (Wong et al., 2011). Here, we showed that PcfF also bends its *oriT* substrate, although seemingly by a mechanism different than TraM. Specifically, in contrast to TraM, which assembles as tetramers in solution, PcfF is dimeric as shown by GEMMA analyses and further supported by the SEC and functional assays. Besides the other evidence, the weak tetramerization interface in the crystal structure only supports a head to tail tetramer, where the two RHH domains sit rotated 180 from each other with the stalk domain in between them (Supplementary Figure S1). This organization of the RHH domains is unlikely to be biologically relevant. The stalk domain, which is responsible for tetramerization of TraM, likely supports only dimerization of PcfF in solution. In our EMSAs, binding induced a DNA shift to a single species with higher molecular mass, as has been shown previously (Chen et al., 2007, 2008), indicating that there is only one DNA binding site per functional PcfF molecule. If PcfF would have been a tetramer, it would have contained two RHH domains and thus be able to bind two independent *oriT* probes in the EMSA. If that was the case one would expect to see a state of two independent retarded species in the EMSA, especially around the concentration corresponding to the apparent dissociation constant (K_D_). Independent of the PcfF concentration used we only observe a single retarded species, indicating that it is the dimeric PcfF that binds to *oriT.* Although we cannot exclude that PcfF can under some circumstances function as a tetramer, e.g., upon relaxosome assembly, our data points toward that the dimeric form of PcfF is the functional unit. In view of these findings, we propose that a single PcfF dimer suffices to bind and bend pCF10’s *oriT* sequence.

Structural and sequence analysis revealed that the stalk domain possesses a conserved sequence motif, NINQ. These residues, together with a few flanking residues (I70, N73, Q74, and R77), form a patch on the surface of PcfF (Supplementary Figure S4). We gained evidence that this patch mediates binding of PcfF to PcfG by showing that a single point mutation (I70S) abolishes PcfF-PcfG binding (Figure 4). Other mutations around this site also completely (N73A/Q74A) or partially (R77S) disrupt this interaction. Finally, PcfF_1–54_, (lacking the entire stalk domain) does not bind PcfG, firmly establishing the importance of the stalk domain for the PcfF-PcfG interaction (Figure 4). In EMSAs, we do not observe any significant additional retarded species upon the addition of PcfG-His to the reaction mix of PcfF-*oriT* or PcfF_1–54_-*oriT*, in contrast to what was previously shown (Chen et al., 2007). We attribute the differences observed between the previous study and our results to different experimental conditions and protein constructs.

Our finding that mutations in, or a complete deletion of, the stalk domain of PcfF attenuates but does not totally abolish pCF10 transfer *in vivo* (Figure 5) indicates that PcfF might be able to indirectly recruit PcfG. We speculate that this could be facilitated via bending and unwinding of the DNA upon PcfF binding. Another accessory factor, TrwA, also binds its cognate *oriT* sequence via an N-terminal RHH domain, whereas its C-terminal domain (which has not been structurally characterized) was shown to not bind to the TrwC relaxase but rather to TrwB (Tato et al., 2007). TrwB is a member of the superfamily of ATPases known as coupling proteins, which are associated with the T4SSs and function in recruitment of cognate substrates for delivery into the transfer channel (Grohmann et al., 2017). Our finding that the PcfF_1–54_ variant supports pCF10 transfer *in vivo* establishes that the stalk domain is not only dispensable for relaxosome assembly but is also not required for docking of the relaxosome with the PcfC coupling protein in *E. faecalis*.

It is more difficult to reconcile the lack of strong effects of the RHH-domain mutations (R13L, R13L/I14A), which abolish PcfF-*oriT* binding *in vitro*, on pCF10 transfer *in vivo* (Figure 5). Equivalent mutations in conserved polar, charged residues in the β-sheets of other RHH accessory factors diminish plasmid transfer by more than 3 orders of magnitude (Moncalian and de la Cruz, 2004; Yoshida et al., 2008; Varsaki et al., 2009), although mutant accessory factors can still support a modest level (10^−5^ – 10^−6^ Tc’s/D) of plasmid transfer. Even though PcfF homodimers stably bind *oriT* sequences *in vitro*, it is possible that additional binding surfaces or residues are exposed when PcfF binds PcfG, which can contribute to *oriT* binding *in vivo*. Alternatively, PcfF might be capable of binding *oriT* secondary structures that form only *in vivo* through surface-exposed residues other than those mutated in our study. Finally, we cannot exclude the possibility that other unidentified plasmid- or host-encoded factors, e.g., IHF-like proteins, contribute to relaxosome assembly *in vivo* (Nelson et al., 1995; Karl et al., 2001). Discriminating between these possibilities will require further investigations.

## Conclusion

In summary, RHH domains of several accessory factors associated with conjugation have now been solved and functionally characterized. However, besides PcfF reported here, only one other structure exists for a full-length accessory factor. The structural basis for PcfF binding to pCF10’s *oriT* sequence resembles that identified for other RHH domain containing proteins. However, in contrast to most other members, PcfF forms dimers instead of tetramers in solution and does not show cooperative binding. Furthermore, mutations in conserved charged, polar residues in the DNA binding b-sheet motif do not block *oriT* substrate binding *in vivo*. We also showed that the α-helical stalk domain contributes to binding of the relaxase PcfG, despite the fact that the dimeric RHH domain alone retains the capacity to orchestrate assembly of the functional relaxosome and support plasmid transfer *in vivo*. Together, our findings underscore both the structural conservation and functional plasticity of accessory factors as nucleators of relaxosome assembly among the conjugation systems.

## Author Contributions

SR, AS, and RB performed the cloning, protein purification and structure determination. SR and AS collected the diffraction data. SR, AS, and LL performed the EMSA experiments and protein size determination via GEMMA and SEC. AS performed the DNA bending experiments. YL performed the cloning in *E. faecalis* and the *in vivo* conjugation experiments. SR, PC, and RB planned the experiments, performed the data analysis and wrote the manuscript with input from all authors.

## Conflict of Interest Statement

The authors declare that the research was conducted in the absence of any commercial or financial relationships that could be construed as a potential conflict of interest.
