# Supplementary material for: Enterococcal PcfF Is a Ribbon-Helix-Helix Protein That Recruits the Relaxase PcfG Through Binding and Bending of the oriT Sequence
Source: Front Microbiol. 2019 May 7;10:958. doi: 10.3389/fmicb.2019.00958 (PMC6514445; doi:10.3389/fmicb.2019.00958)
Supplement: Supplementary file 1 [file Data_Sheet_1.PDF]

Enterococcal PcfF is a Ribbon-Helix-Helix protein that recruits the relaxase PcfG through binding and bending of the *oriT* sequence

Saima Rehman[1], Yang Grace Li[2], Andreas Schmitt[1], Lena Lassinantti[1], Peter J Christie[2] and Ronnie P-A Berntsson[1,3,*]

[1] Department of Medical Biochemistry and Biophysics, Umeå University, SE-90187 Umeå, Sweden

[2] Department of Microbiology and Molecular Genetics, McGovern Medical School, 6431 Fannin St, Houston, Texas 77030, USA

[3] Wallenberg Centre for Molecular Medicine, Umeå University, Umeå, Sweden

* Corresponding author: ronnie.berntsson@umu.se, +46907865235

Supplementary Tables

Table S1. List of strains and plasmids used for constructs design.

**Strain, plasmid, or oligonucleotide**	**Relevant features or sequence (5'-3')**	**Reference(s)**
Strains:		
Escherichia coli		
TOP10 One Shot	Cloning host	Thermo-Fisher
DH5α	Cloning host	Gibco-BRL
BL21(DE3)	Expression host	Thermo-Fisher
Enterococcus faecalis		
CK104	Rif^R, Fus^R	Kristich *et al.* (2005)
OG1ES	Erm^R, $Strep^R$	Staddon *et al.* (2006)
Plasmids:		
pcfG-pInitial	Cloning vector	This study
pcfG-pBXNH3	Sub-cloning and expressing *his$_{10}$-pcfG*	This study
pcfF-pGex-6P-2	pGex-6P-2 expressing *gst-pcfF*	This study
pcfF-pGex-6P-2-1	pGex-6P-2 expressing *gst-pcfF_R13L*	This study
pcfF-pGex-6P-2-2	pGex-6P-2 expressing *gst-pcfF_ I14A*	This study
pcfF-pGex-6P-2-3	pGex-6P-2 expressing *gst-pcfF_R16L*	This study
pcfF-pGex-6P-2-4	pGex-6P-2 expressing *gst-pcfF_ R13L/I14A*	This study
pcfF-pGex-6P-2-5	pGex-6P-2 expressing *gst-pcfF_ R13L/R16L*	This study
pcfF-pGex-6P-2-6	pGex-6P-2 expressing *gst-pcfF_ R13L/I14A/R16L*	This study
pcfF-pGex-6P-2-7	pGex-6P-2 expressing *gst-pcfF_I70S*	This study
pcfF-pGex-6P-2-8	pGex-6P-2 expressing *gst-pcfF_R77S*	This study
pcfF-pGex-6P-2-9	pGex-6P-2 expressing *gst-pcfF_I70S/R77S*	This study
pcfF-pGex-6P-2-10	pGex-6P-2 expressing *gst-pcfF_ N73A/Q74A*	This study
pcfF-pGex-6P-2-11	pGex-6P-2 expressing *gst-pcfF_Q105W*	This study
pcfF-pGex-6P-2-12	pGex-6P-2 expressing *gst-pcfF_Q105A*	This study
pcfF-pGex-6P-2-ΔC55	pGex-6P-2 expressing *gst-pcfF_1-54*	This study
pCF10	Pheromone-inducible conjugative plasmid	Dunny *et al.* (1981)
pCF10ΔpcfF	pCF10 deleted of *pcfF*	Chen et al. (2007)
pCY33	pET-28b(+) expressing *his$_6$-pcfF*	Chen *et al.* (2007)
pYGL194	pET-28b(+) expressing *his$_6$-pcfF_R13L*	This study
pYGL196	pET-28b(+) expressing *his$_6$-pcfF_R13L/I14A*	This study
pYGL197	pET-28b(+) expressing *his$_6$-pcfF_I70S*	This study
pYGL199	pET-28b(+) expressing *his$_6$-pcfF_1-54*	This study
pDL278p23	*L.lactis* promoter p23 cloned into pDL278	Chen *et al.* (2007)
pCY16	pDL278p23 expressing P_{23}-*pcfF*	Chen et al. (2007)
pYGL202	pDL278p23 expressing P_{23}-*pcfF_R13L*	This study
pYGL203	pDL278p23 expressing P_{23}-*pcfF_R13L/I14A*	This study
pYGL204	pDL278p23 expressing P_{23}-*pcfF_1-54*	This study
pYGL205	pDL278p23 expressing P_{23}-*pcfF_I70S*	This study

Table S2. Data collection and refinement statistics.

**Data collection summary**	**PcfF SeMet**
Space group	P 1 21 1
Cell dimensions	
a, b, c (Å)	a = 48.84, b = 61.48, c = 89.1
α, β, γ (°)	α = γ = 90, ß =100.97
Resolution (Å)	37.8 - 1.9 (2.034-1.90)
Completeness (%)	99.89 (85.59)
R_{meas} (%)	6.1 (69.5)
I/σ (I)	16.9 (1.92)
CC(1/2)*	99.9 (65.5)
Redundancy	6.6
No. unique reflections	99336
**Refinement summary**	
Resolution (Å)	47.9 – 1.90
R_{work} (%)	20.2
R_{free} (%)	24.8
Number of atoms	
protein	3633
water	212
B-factors	
protein	73.4
water	62.1
r.m.s. deviations	
Bond lengths (Å)	0.019
Bond angles (°)	1.36
Ramachandran statistics	
outliers (%)	0.0
allowed (%)	0.70
favored (%)	99.3

Table S3. Sequences of *oriT* containing DNA used in this study.

DNA used for EMSA	
Random 44bp DNA	AGTGAACTGAAAGAAGAAACACACGTTGCATGAAGAGCTATATA
oriT ‡ 40bp DNA	TCGCAACATGCTAGCATGTTGCTCCGCTTGCAAAAAGAAA
DNA used for bending studies	
oriT 5'	GCGAAATATTGGTACCCCATGGAATCGAGGGATCCTCTAGTCGCAACATGCT AGCATGTTGCTCCGCTTGCAAAAAGAAAAGTCGACACGCGTAGATCTGCTAG CATCGATCCATGGACT
oriT middle	GCGAAATATTGGTACCCCATGGAATCGAGGGATCCTCTAGTCGCAACATGCT AGCATGTTGCTCCGCTTGCAAAAAGAAAAGTCGACACGCGTAGATCTGCTAG CATCGATCCATGGACT
oriT 3'	GTCTCGAGTTTAAAGATATCCAGCTGCCCGGGAGGCCTTCGCGAAATATTGG TACCCCATGGAATCGAGGGATCCTCTAGTCGCAACATGCTAGCATGTTGCTC CGCTTGCAAAAAGAAA

‡ This segment of DNA contains inverted repeats (IR) and a *nic* region, comprising the minimal *oriT* DNA sequence from *E. faecalis* pCF10, as previously reported in Staddon *et al,* 2006.

Supplementary Figures

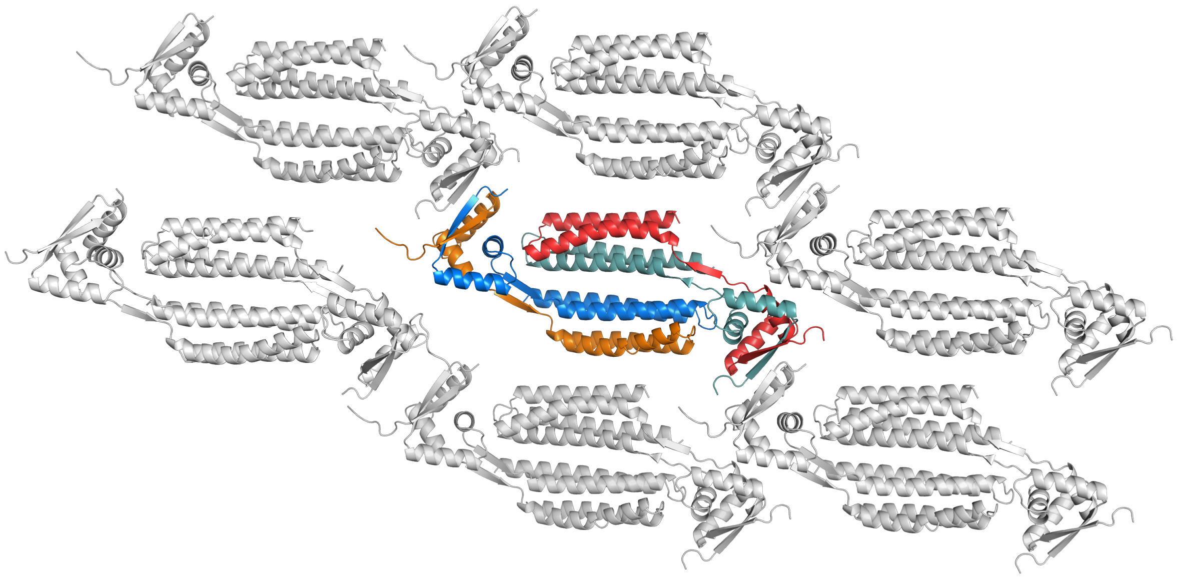

Figure S1. PcfF was crystallized as an antiparallel tetramer (dimer of dimers), here indicated by one dimer in orange and blue, with the second dimer in red and teal. Other symmetry-related tetramers are shown in gray to visualize the crystal packing. The tetrameric interface, which orients the two DNA binding RHH domains at 180° apart, separated by the stalk domain, was deemed unstable by PISA calculations.

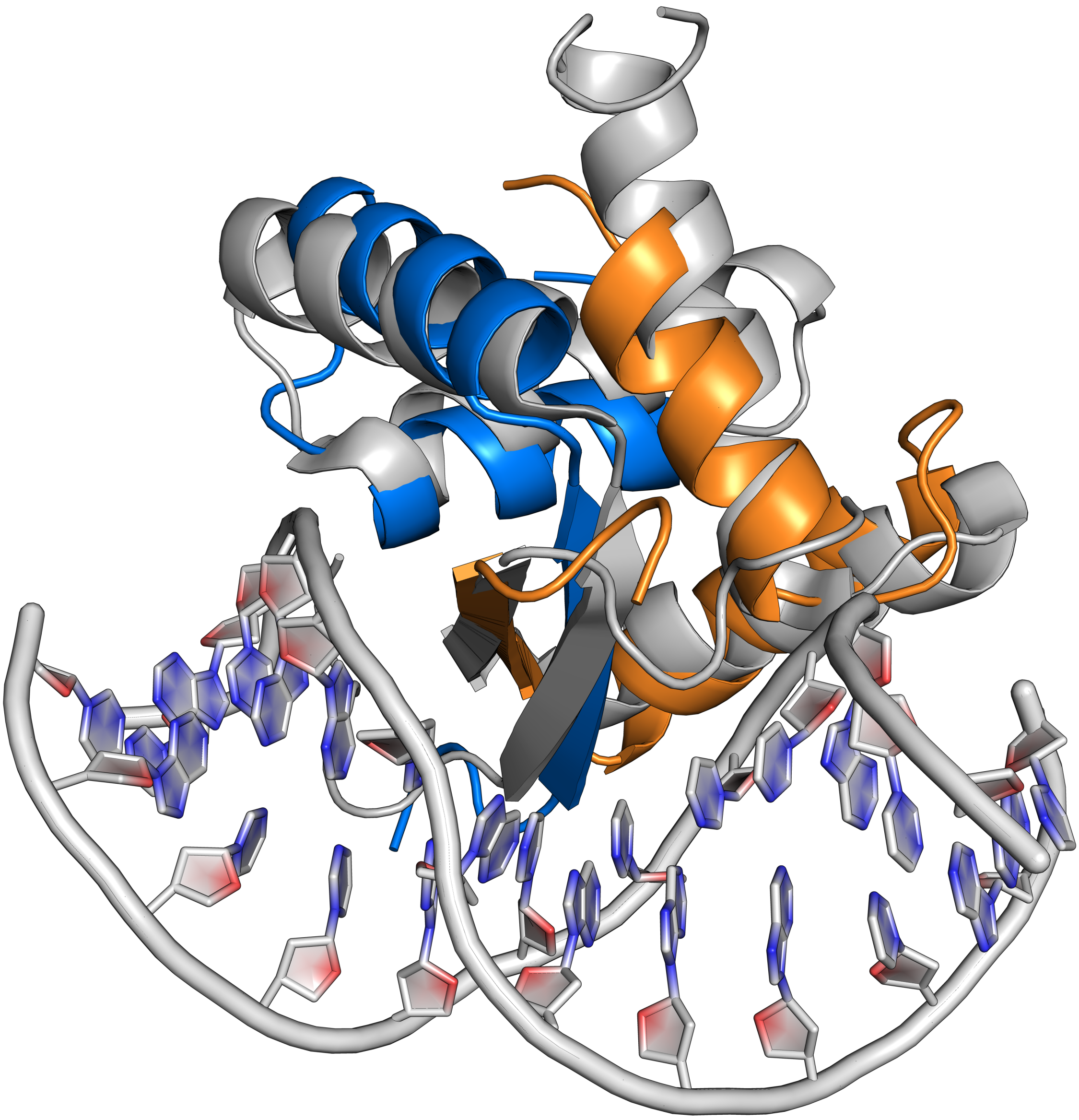

Figure S2.
PcfF's RHH domain (blue and orange) superimposed on the ArcA structure bound to DNA (gray). The RHH domain of PcfF, and especially the N-proximal β-sheet intercalating into the major groove of the DNA, is highly similar to ArcA and superimpose with an R.M.S.D of 2.35 Å.

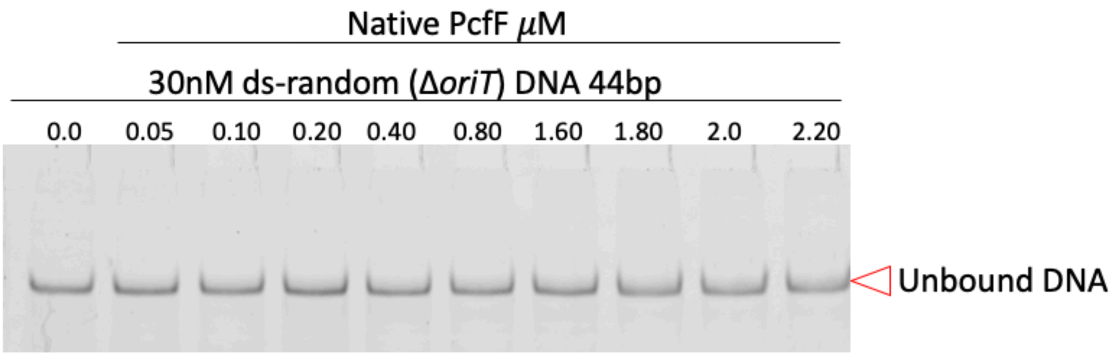

Figure S3. EMSA with a random 44bp long dsDNA (lacking the *oriT* sequence) with increasing concentrations of PcfF. No shift of the DNA is seen even at the highest PcfF concentration.

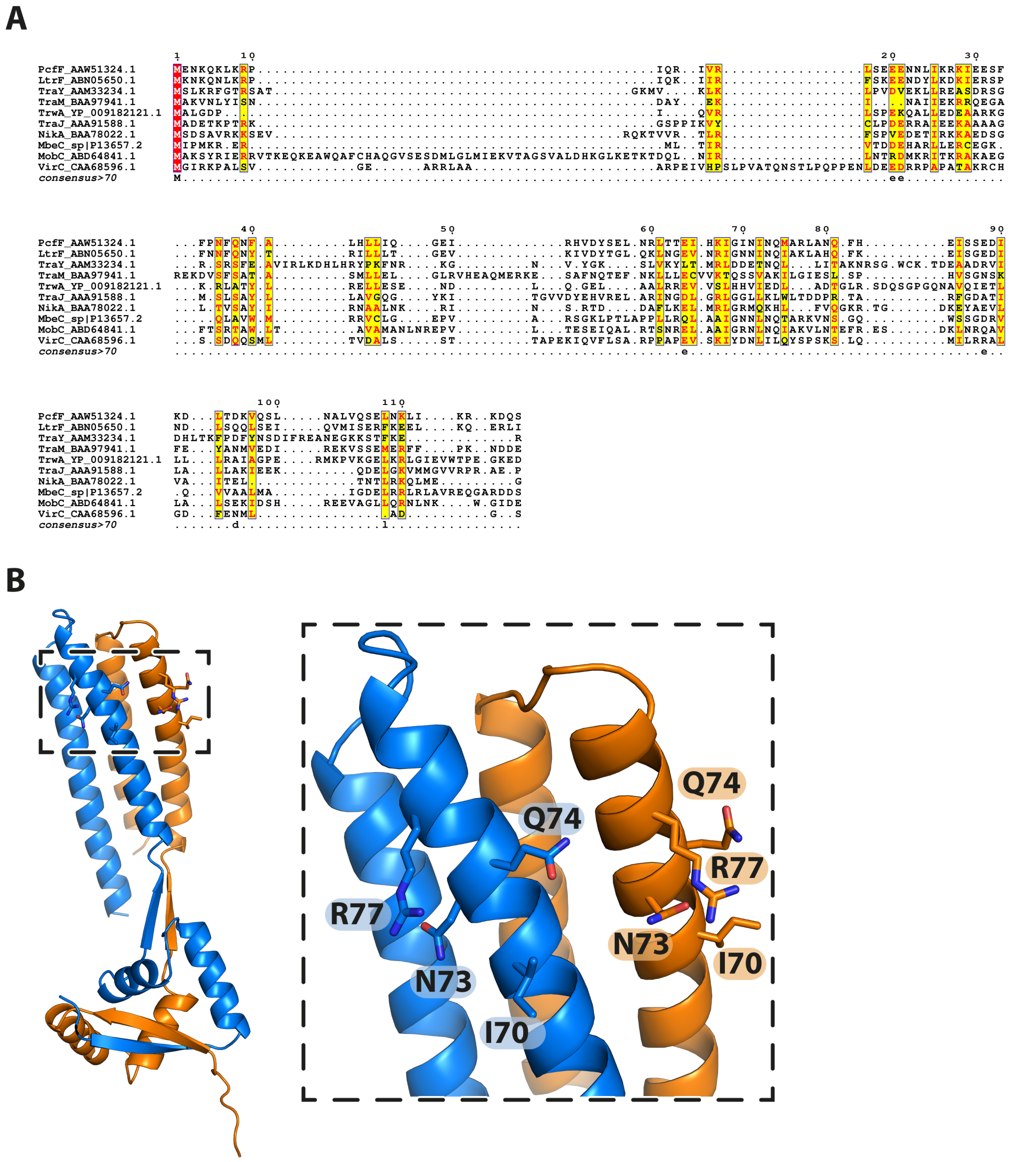

Figure S4. a) Sequence alignment of PcfF and homologs. The NINQ motif (residues 71-74 in PcfF) is semi-conserved among its homologous proteins. Strictly-conserved residues are shown white on red background, text in red with yellow background highlights similarity across groups and lowercase character denotes the consensus residue for consensus level > 0.7. b) Structural overview of the semi-conserved NINQ sequence motif, with the residues mutated in the stalk domain to assay for PcfG interactions highlighted as sticks.

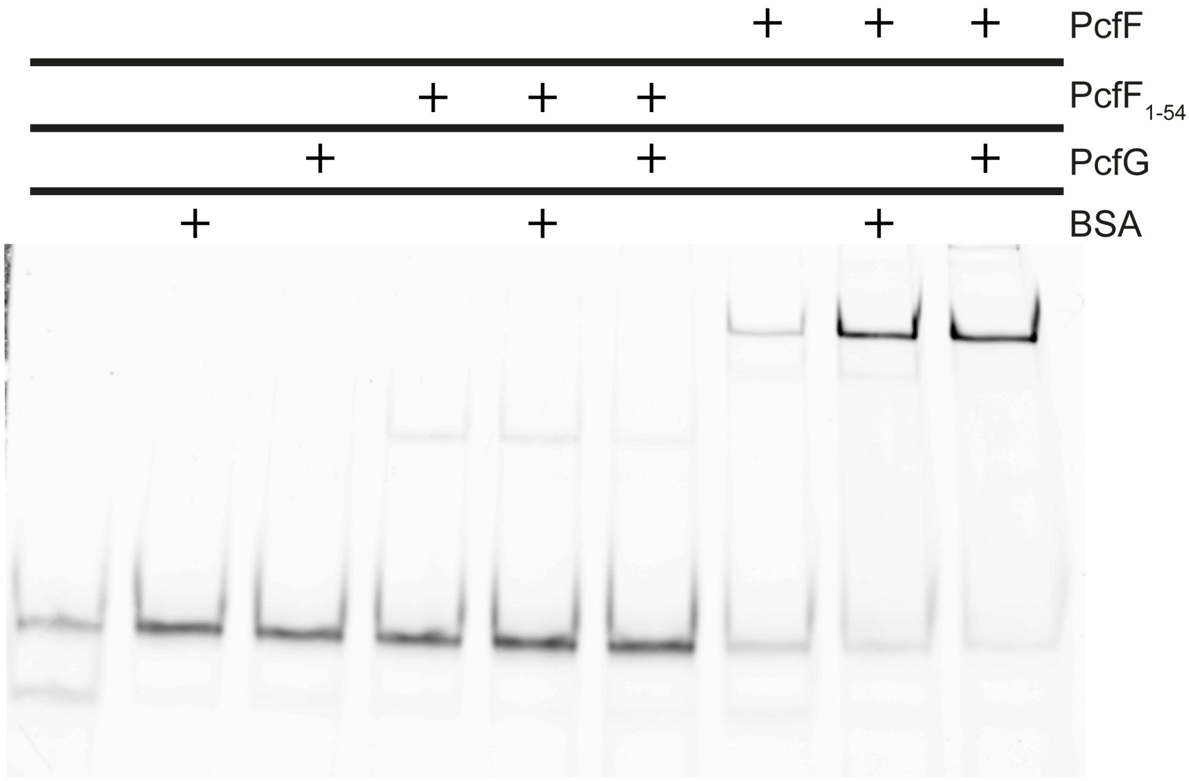

Figure S5. EMSA showing the binding of PcfF, PcfF$_{1\text{-}54}$, and/or PcfG to *oriT,* with BSA used as a control for crowding effect. *oriT* (30 nM) was incubated with PcfF (100 nM), PcfF$_{1\text{-}54}$ (100 nM), PcfG (300 nM) or BSA (300 nM) alone or in combinations. The reactions were analyzed on 20% native polyacrylamide gels. Protein components in each lane are shown on the top of the lane.

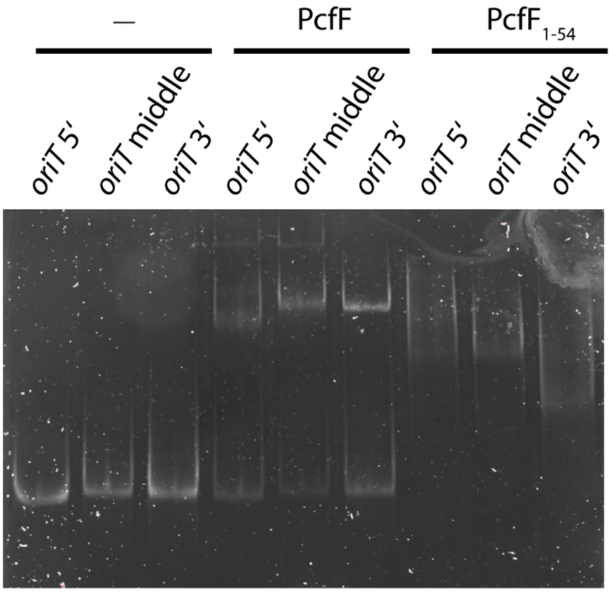

Figure S6. DNA bending assay of *oriT* situated either on the 5', middle or 3' end of a 120 bp long dsDNA. Upon addition of PcfF differential migration of the otherwise equally long DNA molecules are observed, indicating that PcfF bends the DNA upon binding. For $PcfF_{1-54}$ the same experiment always produced smeary bands, making the interpretation more difficult, but a similar trend can be seen.

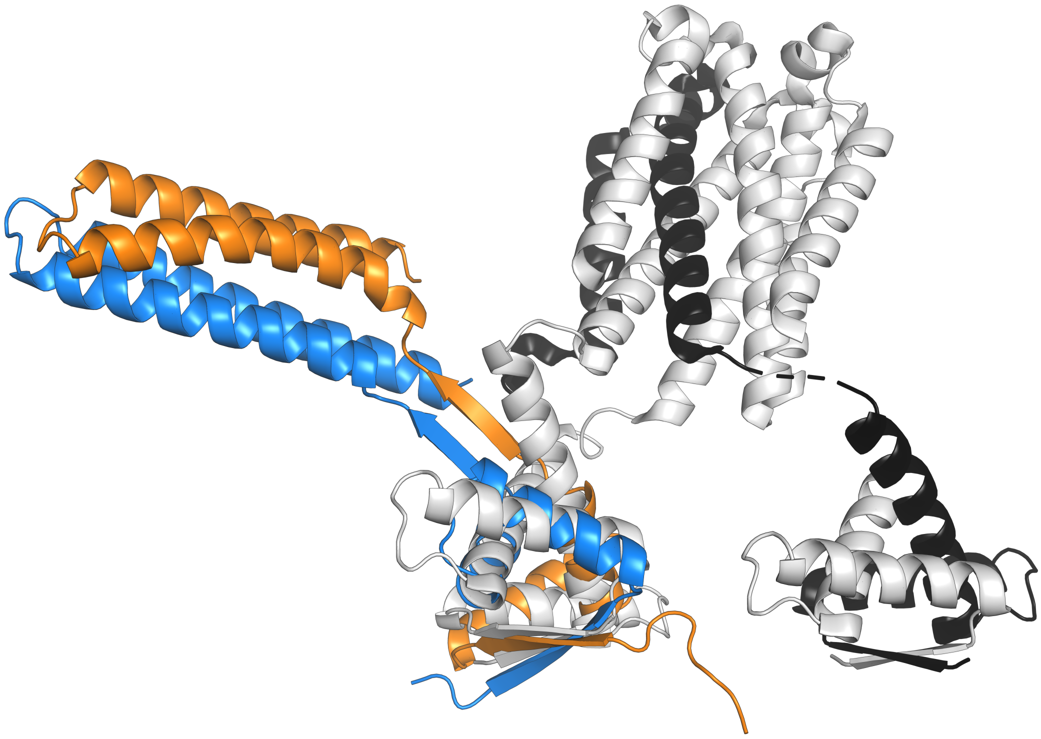

Figure S7. PcfF vs TraM comparison. TraM (gray, PDB code 3ON0) is superimposed on the RHH domain of PcfF (orange and blue). In both proteins, the RHH-domain is made up by a homo-dimer. One of the four chains of TraM is depicted in dark gray to highlight how the stalk domain of TraM acts as a tetramerization domain, where one monomer that forms the right RHH-domain goes up and interacts directly with a monomer from the left RHH-domain.

Supplementary References

Chen, Y., Staddon, J. H., and Dunny, G. M. (2007). Specificity determinants of conjugative DNA processing in the Enterococcus faecalis plasmid pCF10 and the Lactococcus lactis plasmid pRS01. *Mol Microbiol* 63, 1549–1564. doi:10.1111/j.1365-2958.2007.05610.x.

Dunny, G., Funk, C., and Adsit, J. (1981). Direct Stimulation of the Transfer of Antibiotic-Resistance by Sex-Pheromones in Streptococcus-Faecalis. *Plasmid* 6, 270–278.

Kristich, C. J., Manias, D. A., and Dunny, G. M. (2005). Development of a method for markerless genetic exchange in Enterococcus faecalis and its use in construction of a srtA mutant. *Appl Environ Microbiol* 71, 5837–5849. doi:10.1128/AEM.71.10.5837-5849.2005.

Staddon, J. H., Bryan, E. M., Manias, D. A., Chen, Y., and Dunny, G. M. (2006). Genetic characterization of the conjugative DNA processing system of enterococcal plasmid pCF10. *Plasmid* 56, 102–111. doi:10.1016/j.plasmid.2006.05.001.
